# Antibiotic utilization patterns in Tanzania: a retrospective longitudinal study comparing pre- and intra-COVID-19 pandemic era using Tanzania Medicines and Medical Devices Authority data

**DOI:** 10.1093/jacamr/dlae081

**Published:** 2024-05-27

**Authors:** Raphael Z Sangeda, Sahani M William, Faustine C Masatu, Adonis Bitegeko, Yonah H Mwalwisi, Emmanuel A Nkiligi, Pius G Horumpende, Adam M Fimbo

**Affiliations:** Department of Pharmaceutical Microbiology, Muhimbili University of Health and Allied Sciences, P.O. Box 65013, Dar es Salaam, Tanzania; Department of Pharmaceutical Microbiology, Muhimbili University of Health and Allied Sciences, P.O. Box 65013, Dar es Salaam, Tanzania; Medicines Control, Tanzania Medicines and Medical Devices Authority, P.O. Box 1253, Dodoma, Tanzania; Medicines Control, Tanzania Medicines and Medical Devices Authority, P.O. Box 1253, Dodoma, Tanzania; Medicines Control, Tanzania Medicines and Medical Devices Authority, P.O. Box 1253, Dodoma, Tanzania; Medicines Control, Tanzania Medicines and Medical Devices Authority, P.O. Box 1253, Dodoma, Tanzania; Department of Curative Services, Ministry of Health, P.O. Box 743, Dodoma, Tanzania; Department of Biochemistry and Molecular Biology, Kilimanjaro Clinical Research Institute (KCRI), Moshi, Tanzania; Lugalo Infectious Diseases Research Centre, General Military Hospital (GMH) and Military College of Medical Sciences (MCMS), P.O. Box 4000, Dar es Salaam, Tanzania; Medicines Control, Tanzania Medicines and Medical Devices Authority, P.O. Box 1253, Dodoma, Tanzania

## Abstract

**Background:**

Antimicrobial resistance (AMR) is a growing public health concern globally, and misuse of antibiotics is a major contributor.

**Objective:**

This study investigated antibiotic utilization patterns before and during the COVID-19 pandemic in Tanzania using data from the Tanzania Medicines and Medical Devices Authority (TMDA).

**Methods:**

This retrospective longitudinal study analysed secondary data. The study compared antibiotics consumption in defined daily doses per 1000 inhabitants per day (DID) in two distinct eras: 2018–2019 as the pre-COVID-19 era and 2020–2021 as the intra-COVID-19 era. A sample *t*-test was conducted using Statistical Package for the Social Sciences.

**Results:**

The study analysed 10 614 records and found an overall increase in antibiotics consumption from 2018 to 2021. We found that the consumption was 61.24 DID in the intra-COVID-19 era and 50.32 DID in the pre-COVID-19 era. Levofloxacin had the highest percentage increase in use, with a 700% increase in DID during the intra-COVID-19 era. Azithromycin had a 163.79% increase, while cefotaxime had a 600% increase. By contrast, some antibiotics exhibited a decrease in usage during the intra-COVID-19 era, such as nalidixic acid, which had a 100% decrease, and cefpodoxime, which had a 66.67% decrease.

**Conclusions:**

Increased antibiotic consumption during the COVID-19 pandemic highlights the importance of implementing effective antimicrobial stewardship strategies to prevent AMR, especially during pandemics.

## Introduction

Antimicrobial resistance (AMR) poses a serious global health threat by hindering the treatment of bacterial infections.^1^ In low- and middle-income countries (LMICs), including Tanzania, the misuse and overuse of antibiotics have resulted in high rates of AMR, making it challenging to treat bacterial infections.^2,3^

The Global Action Plan for Antimicrobial Resistance aims to address the mounting challenge of increasing AMR through surveillance of antimicrobial use (AMU) and the development of antimicrobial stewardship (AMS) programmes. Consequently, in Tanzania, AMS was introduced through the NAP on AMR^4^ in 2017 and the second version of NAP^5^ 2023–2028 focuses on monitoring AMU in humans and animals.^6^

This study aimed to investigate the changes and trends in antibiotic utilization patterns in Tanzania before and during the intra-COVID-19 eras using Tanzania Medicines and Medical Devices Authority (TMDA) data from 2018 to 2021.

## Methods

### Study design, setting and period

This was a retrospective and longitudinal study conducted in Tanzania Mainland. The importation data was collected from TMDA headquarters in Dodoma, Tanzania, from January 2018 to December 2021.

### Data collection

TMDA has developed and issued regulations and procedures that compel importers to apply for importation permits archived in the Regulatory Information Management System.^6^ The data retrieved included antibiotic descriptions, generic names, strengths, dosage forms, pack sizes, prices, quantities, unit prices and issue dates. The Anatomical Therapeutic Chemical (ATC) classification system and the daily defined dose (DDD), importers and WHO Access, Watch, Reserve (AWaRe) classification (2021) status of antibiotics were also included.^7^ Utilization was expressed in DDD per 1000 inhabitants per day (DID) in accordance with the ATC/DDD (2019) WHO collaborating Center for Statistics Methodology.^8^

### Data analysis

The sample *t*-test was conducted using the Statistical Package for the Social Sciences v.26.0 to assess the impact of the pre- and intra-COVID-19 era on antibiotics consumption. A *P* value of less than 0.05 was considered statistically significant.

### Ethical considerations

Ethical approval (DA. 25/111/28/01/2021) was obtained from the Muhimbili University of Health and Allied Sciences Research Ethics Committee.

## Results

In total, 9610 records of antibiotics imported for systemic use by humans between 2018 and 2021 were retrieved.

A total of 117.02 DID were used in Tanzania between 2018 and 2021, with a mean (standard deviation) of 29.25 (±4.63) DIDs. The year 2021 had the highest DID at 33.1, 47.0% higher than 2019, with the lowest DID at 22.5 (Table S1, available as Supplementary data at *JAC-AMR* Online).

**Figure 1.**
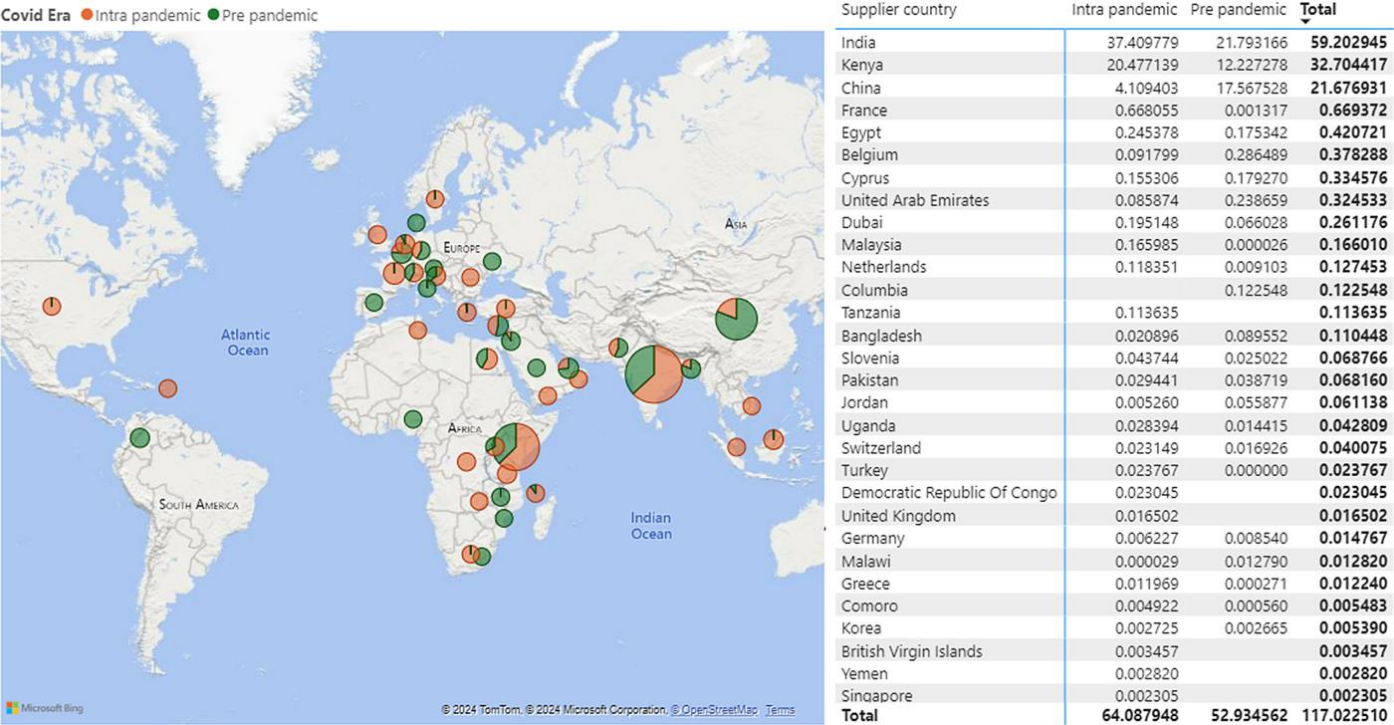
Worldwide country frequency of importation of antibiotics in the pre- and intra-COVID-19 eras. The size of the bubble signifies the DIDs imported from the respective country.

Tanzania imports these antibiotics from across continents and Kenya, India and China were the major sources of antibiotics in the pre- and intra-COVID-19 eras. Tanzania and South Africa were sources of antibiotics only during the intra-COVID-19 era (Figure 1).

The oral dosage form contributed 151.18 (96.93%) of the DIDs (Figure S1). The contribution of individual dosage forms indicated that capsules contributed the most (Figure S2) and (Table S2).

Overall, the Access group had the highest DID at 82.9, followed by Watch, other and Reserve. The Access group accounted for 70.8% (Figure S3) of the DID. The annual increase in the Watch group of antibiotics parallels a general decline in the Access group (Figure S3) and (Figure S4).

Using a paired samples *t-*test, the mean (M) and standard deviation (SD) of antibiotics consumption in the pre-COVID-19 period (M = 1.018, SD = 3.311) was significantly different from the intra-COVID-19 period (M = 1.232, SD = 3.796), *t* (51 = −2.513, *P* = 0.015 and paired sample correlation of 0.994 with effect size, as measured by Cohen's *d*, being 0.312.

Overall, there was a 21% increase in the utilization of antibiotics intra-COVID-19. Azithromycin (J01FA10) increased by 163% during the intra-COVID-19 era (Table S3).

**Figure 2.**
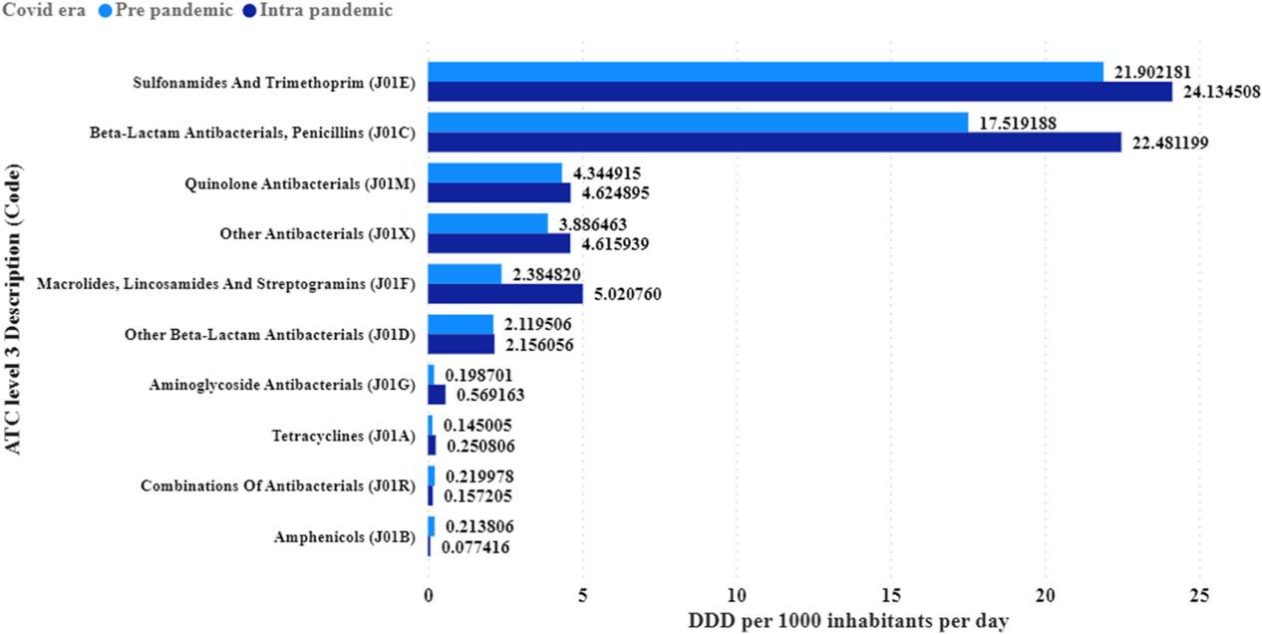
Contribution of each class (level 3 ATC classification) of antibiotics used in Tanzania from 2018 to 2021.

Using level 3 of the ATC classification, beta-lactam antibacterials and penicillins (J01C) registered a significant 28.32% increase in consumption. A 4.96 DID increase between post-COVID and pre-COVID was noted for the beta-lactam antibacterials (Figure 2) and (Table S4).

Aminoglycoside antibacterials (J01G) exhibited a remarkable 186.44% increase. By contrast, amphenicols (J01B) experienced a substantial decrease by 63.79%. The class of macrolides, lincosamides and streptogramins (J01F) flagged a remarkable increase of 110.53% in consumption (Table S4). In addition, annual trends of antibiotics at class 3 of the ATC classification were observed (Table S5), where sulfonamides and trimethoprim (J01E) comprised 20.62% of all DID used, with the highest totals in the pre- and intra-COVID-19 eras. Similar trends were indicated when considering the level 4 ATC classification (Table S6).

## Discussion

We observed an annual increase in the total consumption of antibiotics, reaching 117.02 DID over 4 years. The consumption was 64.09 DID in the intra-COVID-19 era and 52.93 DID in the pre-COVID-19 era. Nevertheless, the mean is 29.25 (±4.01) compared to the mean of 22.07 (±48.85) DID in 2010 to 2016 consumption data in Tanzania.^6^ The 2019 value of utilization is less than previously predicted,^6^ reflecting the impact of AMS under the NAP implementation.^4^

A paired samples *t*-test indicated a statistically significant increase in antibiotics consumption during the intra-COVID-19 era. The effect size, as measured by Cohen's *d*, was 0.312, indicating a small but practically significant increase. The high correlation (*r* = 0.994) between the two eras reinforces the reliability that the COVID-19 pandemic had a notable impact on antibiotics consumption in Tanzania.

The combined use of all antibiotics increased by 21.1% from the pre-COVID-19 period to the intra-COVID-19 period. An increase was noted for gentamicin (J01GB03) at +204.3%, followed by azithromycin (J01FA10) at +163.3% and tetracycline (J01AA07) at +141.2%. A decrease was observed in chloramphenicol (J01BA01) (−64.6%), norfloxacin (J01MA06) (−37.4%) and nitrofurantoin (J01XE01) (−31.1%). A 150% increase in azithromycin use was noted in other studies in LMICS and HICs.^9^ A study in Croatia showed that azithromycin distribution increased from 1.76 in 2017 to 2.01 days of therapy units/1000 inhabitant-days in 2017–2020, indicating azithromycin overuse.^10^ Other reports during the pandemic showed that azithromycin consumption increased up to three times compared to pre-COVID-19.^9,10^

Interestingly, the popularity of azithromycin emerged from reports of its antiviral activity and early pandemic reports of screening indicating potential activity for SARS-CoV-2 alone or in combination with hydroxychloroquine.^11^ Later, several randomized clinical trials suggested that azithromycin does not reduce hospital admissions, respiratory failure or death when compared to conventional therapy and, therefore, azithromycin should no longer be used to treat COVID-19.^12–15^

Several studies have revealed a significant increase in resistance to azithromycin in some strains of *Neisseria gonorrhoeae*, *E. coli* and *Streptococcus pneumoniae*.^11,16–18^ Therefore, continued use of azithromycin should have been limited to infections for which azithromycin is recommended rather than COVID-19.^11^

Examining the consumption at level 3 of ATC classification, we noted a remarkable increase during the intra-COVID-19 era of beta-lactam antibacterials, which penicillins (J01C) and aminoglycoside antibacterials (J01G) exhibited. At the same time, amphenicols (J01B) experienced a substantial decrease of up to −63.79%. The use of macrolides, lincosamides and streptogramins (J01F) also increased remarkably by 110.53%. This finding underscores the specific impact of the pandemic on the consumption of these antibiotics. The major contributor to this increase was azithromycin.

The overall consumption of antibiotics increased from 52.935 DID (pre-COVID-19) to 64.088 DID (intra-COVID-19), with a total change of 21.07%.

Overall, the ATC level 3 class of sulfonamides and trimethoprim (J01E) ranked the top consumed group with only a 10.19% increase in consumption, suggesting continued reliance on this class of antibiotics during the pandemic. This could be due to their effectiveness against certain infections and their wide availability, especially for HIV/AIDS patients. This is usually indicated by the higher contribution of sulfamethoxazole + trimethoprim (J01EE01) used in the HIV programme.

For tetracycline (level 3 class J01A), there was a moderate consumption increase from 0.145 DID (pre-COVID-19) to 0.251 DID (intra-COVID-19, a 72.96% increase, even though this class ranked lower compared to previous studies in Tanzania where the class was among the top contributors of consumed antibiotics.^6^ It is important to note that the percentage change in usage should be taken with caution since it is calculated based on a relatively small difference in values, and the absolute values of DID for each antibiotic may vary significantly.

A recent study conducted in Cameroon during the COVID-19 pandemic revealed that antibiotics were highly overused and misused, leading to increased AMR.^19^

This study is one of the few conducted in sub-Saharan Africa to estimate antibiotics utilization at the national level. The data indicate an increase in the consumption of antibiotics during the intra-COVID-19 era, with a mean of 29.25 DIDs used in Tanzania between 2018 and 2021. This average was less than that studied between 2010 and 2016 in Tanzania, where the mean was 57.4 DIDs over seven years. A study conducted in Tanzania from 2017 to 2019 also reported a slightly higher average compared to this study.^20^

These results highlight the importance of expanding the monitoring of AMU and implementing AMS programmes to address the issue of AMR, especially during global health crises such as the COVID-19 pandemic. The observed changes in antibiotic consumption highlight the need for continued monitoring and the development of interventions to ensure the rational use of antibiotics since the increase in overall consumption may contribute to AMR. Antibiotic stewardship programmes must be emphasized intra-COVID-19 in the healthcare landscape.

According to this AWaRe classification, the Access group consists of antibiotics that are active against many susceptible bacteria and have lower resistance potential than antibiotics in the other groups. In this study, the proportion of Access antibiotics was 82.9%, more than the 60% cutoff suggested by the WHO. The utilization of Watch antibiotics with higher resistance potential is increasing annually. Nevertheless, utilization of Reserve antibiotics for treating infections due to multi-drug-resistant organisms^7^ is minimal.

### Limitations of the study

Our study could not exclude some antibiotics that may have expired or were re-exported to neighbouring countries and those produced locally. Moreover, regional variations in consumption are not accounted for, which is important for understanding local healthcare practices and the impact of AMS interventions.

### Conclusion

This study highlights an increase in the consumption of antibiotics during the COVID-19 pandemic in Tanzania.

## Recommendations

Monitoring AMU in countries via import permits may be a novel way to track this consumption in LMICs. Informative studies using data from community pharmacies and hospitals may provide accurate antibiotic consumption, continuous surveillance and AMS interventions.

## Supplementary Material

dlae081_Supplementary_Data

## Data Availability

All data are included in this article.

## Supplementary data

Figures S1–S4 and Tables S1–S6 are available as Supplementary data at *JAC-AMR* Online.
