# Supplementary material for: Antibiotic utilization patterns in Tanzania: a retrospective longitudinal study comparing pre- and intra-COVID-19 pandemic era using Tanzania Medicines and Medical Devices Authority data
Source: JAC Antimicrob Resist. 2024 May 27;6(3):dlae081. doi: 10.1093/jacamr/dlae081 (PMC11128939; doi:10.1093/jacamr/dlae081)
Supplement: dlae081_Supplementary_Data [file dlae081_supplementary_data.docx]

**Supplementary Figures and Tables**

**Antibiotic Utilisation Patterns in Tanzania: A Retrospective Longitudinal Study Comparing Pre-and Intra-COVID-19 Pandemic Era Using Tanzania Medicines and Medical Devices Authority Data**

**Raphael Z. SANGEDA^1^*, Sahani M. WILLIAM^1^, Faustine Cassian MASATU^2^, Adonis BITEGEKO^2^, Yonah Hebron MWALWISI^2^, Emmanuel Alphonse NKILIGI^2^, Pius Gerald HORUMPENDE^3,4,5^, Adam M. FIMBO^2^**

^1^Department of Pharmaceutical Microbiology, Muhimbili University of Health and Allied Sciences, P.O. Box 65013, Dar es Salaam, Tanzania

^2^Tanzania Medicines and Medical Devices Authority, P.O. Box 1253, Dodoma, Tanzania

^3^Department of Curative Services, Ministry of Health, P.O. Box 743 Dodoma, Tanzania.

College, Moshi, Tanzania

^4^Kilimanjaro Clinical Research Institute (KCRI), Moshi, Tanzania

^5^Lugalo Infectious Diseases Research Centre, General Military Hospital (GMH) and Military College of Medical Sciences (MCMS), P.O. Box 4000 Dar es Salaam, Tanzania

^*^Correspondence to Raphael Zozimus Sangeda email: [sangeda@gmail.com](mailto:sangeda@gmail.com)

**Short title: Influence of COVID-19 on Antibiotic Utilization Patterns in Tanzania**

**Table S1: Annual distribution of DIDs and number of permits of antibiotics imported in Tanzania between 2018 and 2021**

| **Year** | **DID** | **Number of Permits** |
| --- | --- | --- |
| **2018** | 30.39831 | 2,491 |
| **2019** | 22.53625 | 2,426 |
| **2020** | 30.96806 | 2,152 |
| **2021** | 33.11989 | 2,541 |
| **Total** | **117.0225** | **9,610** |

Key: DID: Daily Defined Dose per 1000 inhabitants per day


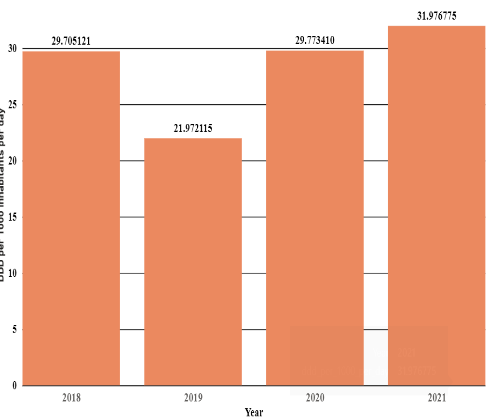

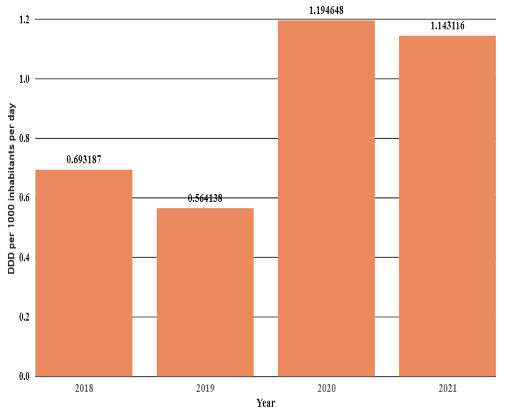


A

B

***Figure S1: DID contribution for oral (Panel A) and parenteral (panel B) antibiotics***


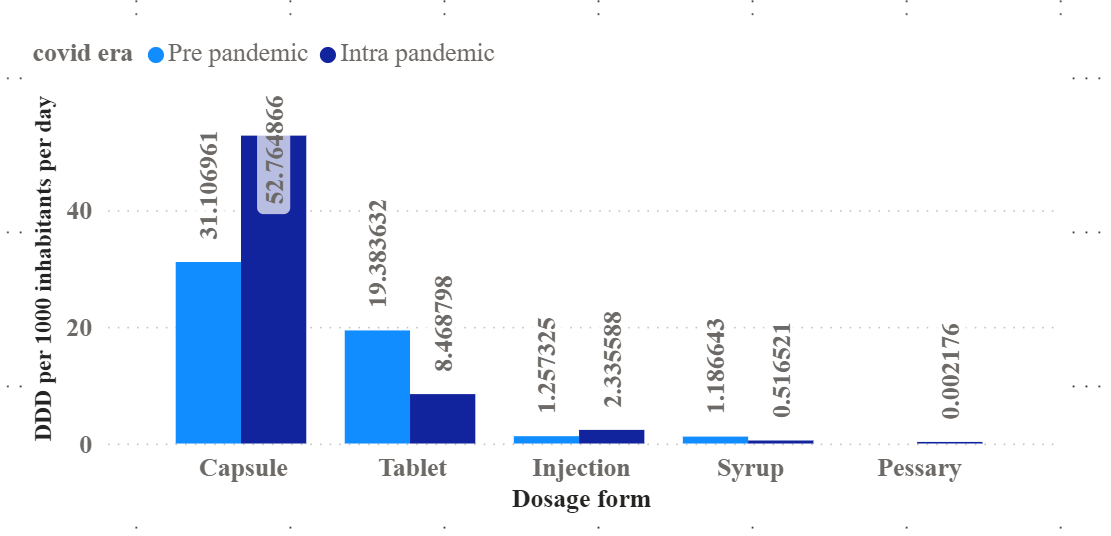


**Figure S2: Contribution of antibiotics consumption per dosage and by COVID-19 era**

**Table S2: Annual contribution of consumption of antibiotics per dosage form**

|  | **Year** | **% contribution** | | | |
| --- | --- | --- | --- | --- | --- |
|  |  |  | | | |
| **Dosage form** | **2018** | **2019** | **2020** | **2021** | **All time** |
| Capsules | 52.5 | 67.2 | 84.7 | 80.2 | 71.1 |
| Injections | 2.3 | 2.5 | 3.9 | 3.4 | 3.0 |
| Pessaries |  |  |  | 0.0 | 0.0 |
| Syrup | 2.7 | 1.6 | 0.7 | 0.9 | 1.5 |
| Tablets | 42.5 | 28.7 | 10.8 | 15.5 | 24.4 |
| **Total** | **100.0** | **100.0** | **100.0** | **100.0** | 100.0 |


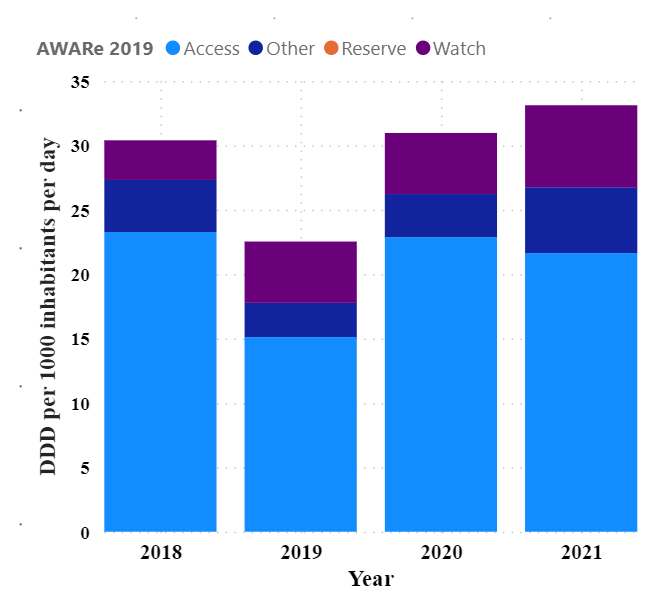

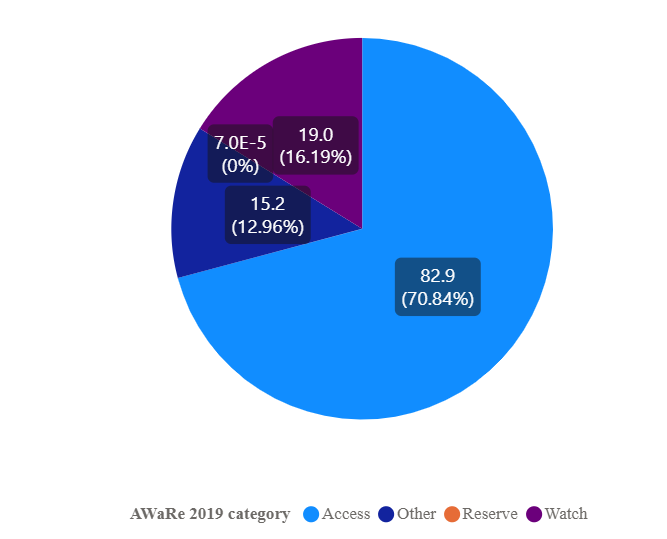


**A**

**B**

***Figure S3: DID contribution per WHO AWaRe classification of antibiotics consumption from 2018-2021 (Panel ) and overall for four years (panel b)***

*
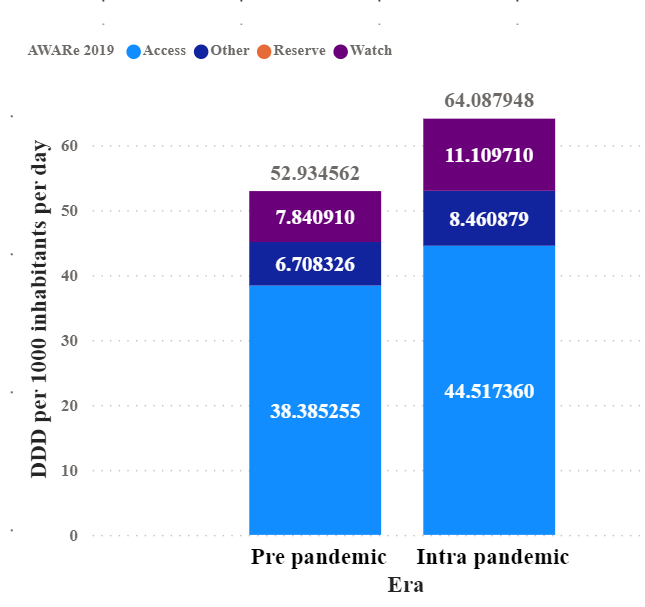
*

***Figure S4: Distribution of Defined Daily Dose (DDD per 1000 inhabitants per day (DID)) of antibiotics per the World Health Organization's AWaRe class for antibiotics utilised in Tanzania from 2018 to 2020.***

***Table S3: Percentage changes in consumption during COVID-19 for top 20 consumed antibiotics aggregated per level 5 WHO ATC classification in DID***

|  | **DID** | | | **% Change** |
| --- | --- | --- | --- | --- |
| **Antibiotic (ATC level 5 code)** | **Pre COVID-19** | **Intra COVID-19** | **Total** |  |
| Sulfamethoxazole + Trimethoprim (J01EE01) | 21.90206 | 24.134214 | 46.036274 | 10.2 |
| Amoxicillin (J01CA04) | 8.958047 | 12.109729 | 21.067776 | 35.2 |
| Ampicillin + Cloxacillin (J01CR50) | 4.957764 | 6.056899 | 11.014663 | 22.2 |
| Ciprofloxacin (J01MA02) | 3.320329 | 3.875245 | 7.195574 | 16.7 |
| Metronidazole (J01XD01) | 2.550591 | 3.007772 | 5.558363 | 17.9 |
| Azithromycin (J01FA10) | 1.163502 | 3.063967 | 4.227469 | 163.3 |
| Phenoxy methyl Penicillin (J01CE02) | 1.839013 | 2.112984 | 3.951997 | 14.9 |
| Erythromycin (J01FA01) | 1.103971 | 1.837235 | 2.941206 | 66.4 |
| Tinidazole (J01XD02) | 0.988315 | 1.399698 | 2.388013 | 41.6 |
| Amoxicillin + Clavulanate (J01CR02) | 0.81814 | 1.280495 | 2.098635 | 56.5 |
| Ceftriaxone (J01DD04) | 0.912814 | 1.147269 | 2.060083 | 25.7 |
| Cefalexin (J01DB01) | 0.956694 | 0.637602 | 1.594296 | -33.4 |
| Norfloxacin (J01MA06) | 0.929865 | 0.582542 | 1.512407 | -37.4 |
| Ampicillin (J01CA01) | 0.737038 | 0.699213 | 1.436251 | -5.1 |
| Gentamicin (J01GB03) | 0.184052 | 0.560144 | 0.744196 | 204.3 |
| Amoxicillin + Flucloxacillin (J01CR50) | 0.1963 | 0.216417 | 0.412717 | 10.2 |
| Ciprofloxacin + Tinidazole (J01RA11) | 0.219978 | 0.157204 | 0.377182 | -28.5 |
| Nitrofurantoin (J01XE01) | 0.220675 | 0.152053 | 0.372728 | -31.1 |
| Tetracycline (J01AA07) | 0.09207 | 0.222064 | 0.314134 | 141.2 |
| Chloramphenicol (J01BA01) | 0.210731 | 0.074664 | 0.285395 | -64.6 |
| Cefixime (J01DD08) | 0.089679 | 0.18745 | 0.277129 | 109.0 |
| Clarithromycin (J01FA09) | 0.116368 | 0.116796 | 0.233164 | 0.4 |
| Ornidazole (J01XD03) | 0.124153 | 0.053845 | 0.177998 | -56.6 |
| Cefuroxime (J01DC02) | 0.073682 | 0.04626 | 0.119942 | -37.2 |
| Ofloxacin (J01MA01) | 0.052129 | 0.06318 | 0.115309 | 21.2 |
| Cefadroxil (J01DB05) | 0.041038 | 0.050992 | 0.09203 | 24.3 |
| Levofloxacin (J01MA13) | 0.008836 | 0.082444 | 0.09128 | 833.0 |
| Doxycycline (J01AA02) | 0.052935 | 0.028352 | 0.081287 | -46.4 |
| Cefotaxime (J01DD01) | 0.005643 | 0.066155 | 0.071798 | 1072.3 |
| Cefpodoxime (J01DD13) | 0.02914 | 0.006786 | 0.035926 | -76.7 |
| Moxifloxacin (J01MA15) | 0.015046 | 0.012868 | 0.027914 | -14.5 |
| Dexamethasone + Neomycin + Polymyxin B (J01GB05) | 0.014225 | 0.008568 | 0.022793 | -39.8 |
| Lomefloxacin (J01MA07) | 0.010553 | 0.008616 | 0.019169 | -18.4 |
| Meropenem (J01DH02) | 0.00761 | 0.009849 | 0.017459 | 29.4 |
| Ampicillin + enzyme inhibitor (J01CA51) | 0.011029 | 0.004753 | 0.015782 | -56.9 |
| Nalidixic Acid (J01MB02) | 0.008157 |  | 0.008157 | -100.0 |
| Lignocaine + Chloramphenicol + Beclomethasone Dipropiote + Clotrimazole (J01BA01) | 0.003076 | 0.002752 | 0.005828 | -10.5 |
| Clindamycin (J01FF01) | 0.00078 | 0.002762 | 0.003542 | 254.1 |
| Spectinomycin (J01XX04) | 0.001507 | 0.00086 | 0.002367 | -42.9 |
| Cefepime (J01DE01) | 0.001017 | 0.000937 | 0.001954 | -7.9 |
| Flucloxacillin (J01CF05) | 0.001857 |  | 0.001857 | -100.0 |
| Vancomycin (J01XA01) | 0.000392 | 0.001189 | 0.001581 | 203.3 |
| Cefoperazone + combinations (J01DD62) | 0.000996 | 0.000461 | 0.001457 | -53.7 |
| Cefoperazone + Sulbactam (J01DD62) | 0.000497 | 0.000615 | 0.001112 | 23.7 |
| Bacitracin + Neomycin + Polymyxin B (J01XX10 ) | 0.000561 | 0.000423 | 0.000984 | -24.6 |
| Cefpirome (J01DE02) |  | 0.000867 | 0.000867 | NA |
| Cilastatin + Imipenem (J01DH51) | 0.0003 | 0.000487 | 0.000787 | 62.3 |
| Cloxacillin (J01CF02) |  | 0.000709 | 0.000709 | NA |
| Amikacin (J01GB06) | 0.00027 | 0.000181 | 0.000451 | -33.0 |
| Cefazolin (J01DB04) | 0.000174 | 0.000275 | 0.000449 | 58.0 |
| Tylosin Tartrate + Doxycycline Hyclate (J01AA02) |  | 0.000389 | 0.000389 | NA |
| PolyMyxin B (J01XB02) | 0.000199 | 0.0001 | 0.000299 | -49.7 |
| Neomycin (J01GB05) |  | 0.000267 | 0.000267 | NA |
| Roxithromycin (J01FA06) | 0.000199 |  | 0.000199 | -100.0 |
| Sulfadiazine + Trimethoprim (J01EE02) | 0.000051 | 0.000136 | 0.000187 | 166.7 |
| Kanamycin (J01GB04) | 0.000153 |  | 0.000153 | -100.0 |
| Sulfadimidine (J01EB03) |  | 0.000123 | 0.000123 | NA |
| Trimethoprim (J01EA01) | 0.00007 | 0.000035 | 0.000105 | -50.0 |
| Cefaclor (J01DC04) | 0.000077 | 0.000027 | 0.000104 | -64.9 |
| Imipenem + enzyme inhibitor (J01DH56) | 0.000089 |  | 0.000089 | -100.0 |
| Ceftazidime (J01DD02) | 0.000056 | 0.000024 | 0.00008 | -57.1 |
| Linezolid (J01XX08) | 0.00007 |  | 0.00007 | -100.0 |
| Tobramycin (J01GB01) | 0.000001 | 0.000002 | 0.000003 | 100.0 |
| Isoniazid + Pyridoxine + Sulfamethoxazole + Trimethoprim (J04AM08) |  | 0.000001 | 0.000001 | NA |
| Ampicillin + Sulbactam (J01CR01) | 0 |  | 0 | NA |
| Azithromycin + fluconazole + secnidazole (J01RA07) |  | 0 | 0 | NA |
| Erythromycin + combinations (J01FA01) |  | 0 | 0 | N.A. |
| **Period Total** | **52.934564** | **64.087946** | **117.02251** | 21.1 |

**Table S4: Consumption aggregated at ATC level 3 in the pre-COVID-19 and intra-COVID-19 era in Tanzania**

| **ATC level 3 Description (Code)** | **Pre COVID-19** | **Intra COVID-19** | **Class Total** | **Change %** |
| --- | --- | --- | --- | --- |
| Sulfonamides And Trimethoprim (J01E) | 21.902181 | 24.134508 | 46.036689 | 10.19226 |
| Beta-Lactam Antibacterials, Penicillins (J01C) | 17.519188 | 22.481199 | 40.000387 | 28.32329 |
| Quinolone Antibacterials (J01M) | 4.344915 | 4.624895 | 8.96981 | 6.443854 |
| Other Antibacterials (J01X) | 3.886463 | 4.615939 | 8.502402 | 18.76966 |
| Macrolides, Lincosamides And Streptogramins (J01F) | 2.38482 | 5.02076 | 7.40558 | 110.5299 |
| Other Beta-Lactam Antibacterials (J01D) | 2.119506 | 2.156056 | 4.275562 | 1.724458 |
| Aminoglycoside Antibacterials (J01G) | 0.198701 | 0.569163 | 0.767864 | 186.4419 |
| Tetracyclines (J01A) | 0.145005 | 0.250806 | 0.395811 | 72.96369 |
| Combinations Of Antibacterials (J01R) | 0.219978 | 0.157205 | 0.377183 | -28.536 |
| Amphenicols (J01B) | 0.213806 | 0.077416 | 0.291222 | -63.7915 |
| **Period Total** | **52.934563** | **64.087947** | **117.02251** | **21.07014** |

**Table S5: Consumption aggregated at ATC level 3 from 2018 to 2021 in Tanzania**

| **ATC Class level 3** | **2018** | **2019** | **2020** | **2021** | **Four Years Total** |
| --- | --- | --- | --- | --- | --- |
| Sulfonamides And Trimethoprim (J01E) | 14.617083 | 7.285097 | 12.50324 | 11.631268 | 46.036688 |
| Beta-Lactam Antibacterials, Penicillins (J01C) | 10.173568 | 7.34562 | 11.12656 | 11.35464 | 40.000388 |
| Quinolone Antibacterials (J01M) | 1.462383 | 2.882532 | 2.309063 | 2.315832 | 8.96981 |
| Other Antibacterials (J01X) | 1.784649 | 2.101814 | 1.960377 | 2.655562 | 8.502402 |
| Macrolides, Lincosamides And Streptogramins (J01F) | 1.064215 | 1.320605 | 1.65612 | 3.364641 | 7.405581 |
| Other Beta-Lactam Antibacterials (J01D) | 0.872695 | 1.24681 | 1.157145 | 0.998911 | 4.275561 |
| Aminoglycoside Antibacterials (J01G) | 0.155795 | 0.042906 | 0.07892 | 0.490243 | 0.767864 |
| Tetracyclines (J01A) | 0.042675 | 0.10233 | 0.10106 | 0.149746 | 0.395811 |
| Combinations Of Antibacterials (J01R) | 0.060057 | 0.159921 | 0.024369 | 0.132836 | 0.377183 |
| Amphenicols (J01B) | 0.165188 | 0.048619 | 0.051204 | 0.026212 | 0.291223 |
| Drugs For Treatment Of Tuberculosis (J04A) |  |  |  | 0.000001 | 0.000001 |
| **Year Total** | **30.398308** | **22.536254** | **30.968058** | **33.119892** | **117.022512** |

**Table S6: Consumption aggregated at ATC level 4 from 2018 to 2021 in Tanzania.**

| **ATC Class level 4** | **2018** | **2019** | **2020** | **2021** | **Four years Total** |
| --- | --- | --- | --- | --- | --- |
| Amphenicols (J01BA) | 0.165188 | 0.048619 | 0.051204 | 0.026212 | 0.291223 |
| Beta-Lactamase Resistant Penicillins (J01CF) | 0.000632 | 0.001225 | 0.000709 |  | 0.002566 |
| Beta-Lactamase Sensitive Penicillins (J01CE) | 0.97563 | 0.863383 | 1.066293 | 1.04669 | 3.951996 |
| Carbapenems (J01DH) | 0.006551 | 0.001447 | 0.00222 | 0.008116 | 0.018334 |
| Combinations Of Antibacterials (J01RA) | 0.060057 | 0.159921 | 0.024369 | 0.132836 | 0.377183 |
| Combinations Of Drugs For Treatment Of Tuberculosis (J04AM) |  |  |  | 0.000001 | 0.000001 |
| Combinations Of Penicillins, Incl. Beta-Lactamase Inhibitors (J01CR) | 3.626861 | 2.345343 | 3.431805 | 4.122006 | 13.526015 |
| Combinations Of Sulphonamides And Trimethoprim Incl. Derivatives (J01EE) | 14.617026 | 7.285085 | 12.503105 | 11.631245 | 46.036461 |
| First Generation Cephalosporins (J01DB) | 0.310298 | 0.687607 | 0.376731 | 0.312137 | 1.686773 |
| Fluoroquinolones (J01MA) | 1.454226 | 2.882532 | 2.309063 | 2.315832 | 8.961653 |
| Fourth Generation Cephalosporins (J01DE) | 0.000656 | 0.000361 | 0.00063 | 0.001174 | 0.002821 |
| Glycopeptide Antibacterials (J01XA) | 0.000293 | 0.000098 | 0.000166 | 0.001022 | 0.001579 |
| Imidazole Derivatives (J01XD) | 1.710669 | 1.952389 | 1.893326 | 2.567989 | 8.124373 |
| Lincosamides (J01FF) | 0.000276 | 0.000503 | 0.000896 | 0.001866 | 0.003541 |
| Macrolides (J01FA) | 1.063939 | 1.320101 | 1.655224 | 3.362775 | 7.402039 |
| Other Aminoglycosides (J01GB) | 0.155795 | 0.042906 | 0.07892 | 0.490243 | 0.767864 |
| Other Antibacterials (J01XX) | 0.001017 | 0.001122 | 0 | 0.001283 | 0.003422 |
| Other Quinolones (J01MB) | 0.008157 |  |  |  | 0.008157 |
| Penicillins With Extended Spectrum (J01CA) | 5.570445 | 4.135668 | 6.627752 | 6.185944 | 22.519809 |
| Second Generation Cephalosporins (J01DC) | 0.042137 | 0.031622 | 0.009737 | 0.03655 | 0.120046 |
| Short-Acting Sulfonamides (J01EB) |  |  | 0.000123 |  | 0.000123 |
| Tetracyclines (J01AA) | 0.042675 | 0.10233 | 0.10106 | 0.149746 | 0.395811 |
| Third Generation Cephalosporins (J01DD) | 0.513052 | 0.525773 | 0.767827 | 0.640932 | 2.447584 |
| (Not classified) | 0.072727 | 0.148216 | 0.066897 | 0.085291 | 0.373131 |
| **Year Total** | **30.398307** | **22.536251** | **30.968057** | **33.11989** | **117.022505** |
